# Highly Enhancing Electrical, Thermal, and Mechanical Properties of Polypropylene/Graphite Intercalation Compound Composites by In Situ Expansion during Melt Mixing

**DOI:** 10.3390/polym13183095

**Published:** 2021-09-14

**Authors:** Zhifeng Wang, Jun Tong, Wei Li, Haichen Zhang, Manfeng Hu, Haichu Chen, Hui He

**Affiliations:** 1 School of Mechatronic Engineering and Automation, Foshan University, Foshan 528000, China; zhifengw@fosu.edu.cn (Z.W.); liweigdut2007@163.com (W.L.); manfenghu@fosu.edu.cn (M.H.); chenhaichu@fosu.edu.cn (H.C.); 2 School of Materials Science and Engineering, South China University of Technology, Guangzhou 510641, China; 3 School of Materials Science and Hydrogen Energy, Foshan University, Foshan 528000, China; hczhang@fosu.edu.cn; 4 Guangdong Key Laboratory for Hydrogen Energy, Foshan 528000, China

**Keywords:** polymer matrix composites (PMCs), electrical properties, thermal properties, mechanical properties

## Abstract

Polypropylene/graphite intercalation compound (PP/GIC) composites are prepared via melt mixing at three different temperatures (180, 200, and 220 °C). The dispersion of GICs in the composites is clearly improved due to the increased interlamellar spacing caused by in situ expansion of GICs at higher temperatures, which facilitates the intercalation of PP molecular chains into the GIC galleries. As a result, the PP/GIC composite with 10 wt% GICs prepared at 220 °C (PG220) presents a dielectric constant of about 1.3 × 10^8^ at 10^3^ Hz, which is about six orders higher than that of the composite prepared at 180 °C (PG180). Moreover, the thermal conductivity of the PG220 sample (0.63 Wm^−1^K^−1^) is 61.5% higher than that of the PG180 sample. The well-dispersed GICs accelerates the crystallization of PP by increasing the nucleation point and enhances the thermal stability of the composites. The PG220 sample shows a Young’s modulus that is about 21.2% higher than that of the PG180 samples. The results provide an efficient approach for fabricating polymer/GIC composites without complex exfoliation and dispersion processes.

## 1. Introduction

Polymer/conductive filler composites present great application prospects in fields such as electronics, electrical energy conversion, and storage materials due to their light weight and good processability [1,2,3]. Generally, most polymers struggle to meet application demands due to their absence of satisfactory electrical, thermal, and mechanical properties. Therefore, introducing functional carbon fillers, such as expanded graphite (EG) [4,5,6,7], graphene [8,9,10,11], and carbon nanotube [12,13,14,15], is proposed to develop materials with advanced properties. Recently, EG prepared from graphite intercalation compounds (GICs) via rapid heating has been widely selected as a candidate for electrical, thermal, and mechanical properties improvements due to its low cost and high electrical and thermal conductivities. In order to significantly improve the properties of the composites, good dispersion of the EG must be achieved [16]. However, it is difficult to exfoliate and disperse EG in polymer matrices due to the van der Waals force between graphite layers.

In situ polymerization and solution mixing are commonly used methods of dispersing EG in polymer matrices. Chen et al. [17] dispersed non-covalent functionalized EG in poly(methyl methacrylate) via in situ polymerization. The EG is well-dispersed and the composite exhibits a low percolation threshold of 0.31 vol%. Yu et al. [18] reported that dispersing EG into epoxy by bath sonication for 24 h could significantly improve the dispersion state of EG in epoxy matrix and increase the thermal conductivity of the composite up to 6.44 Wm^−1^K^−1^ at an EG content of 25 vol%. Melt mixing is deemed to be an environmentally friendly method for polymer/EG composite preparation [19,20], which avoids the use of organic chemical solutions. However, large EG aggregates tend to be formed in polymer matrices during melt mixing, which can seriously compromise the performances of the composites. Pretreatment of EG is usually required to improve its dispersion state in polymer matrices. Fu et al. [6] prepared poly(vinylidene fluoride) (PVDF)/EG composites with a good dispersion state by pretreating EG via ball milling before melt mixing. As a result, the EG was well exfoliated and dispersed in the PVDF matrix, leading to significantly improved thermal conductivity of the composites. Ultrasound-assisted processing was also used to improve the dispersion of EG in the polymer matrix during melt extrusion. Yang et al. [21] prepared polyamide 6/EG composites via preliminarily exfoliating EG and melt extrusion with ultrasound-assisted mixing. The dispersion of EG in the composites was clearly improved, which led to significantly enhanced mechanical property of the composites. However, the introduction of a pretreatment led to a complex process for composite preparation.

In the authors’ previous work [22,23], PVDF/EG composites were prepared by melt mixing PVDF and EG with water injection. The dispersion of EG in the PVDF matrix was notably improved. As a result, the electrical and thermal conductivities of the composites were enhanced. As EG is prepared by expanding original graphite intercalation compounds (GICs) in a muffle furnace at 600 °C, it exhibits a large, expanded volume, which is not conducive to feeding the EG into the extruder. Generally, GICs can be expanded up to dozens of times at a volume above 200 °C. Thus, GICs can be directly added to polymers by melt mixing. It is expected that the in situ thermal expansion of GICs would be in favor of the intercalation of polymer molecular chains into the layers of GICs during melt mixing. Polypropylene (PP), one of the most common polyolefins due to its low cost, recyclability, and good processability, was chosen in this work. Three different processing temperatures were implemented to investigate the effect of processing temperatures on the exfoliation and dispersion of GICs in the PP matrix and then on the dielectric, thermal, and mechanical properties of the PP/GIC composites.

## 2. Materials and Methods

### 2.1. Materials

PP resin (T30S, melt index: 3.0 g/10 min (2.16 kg/230 °C)) and graphite intercalation compounds (GICs, Nanjing XFNANO Materials Tech Co. Ltd., Nanjing, China) with a particle size of about 300 µm and initial expansion temperature of 200 °C were used as received.

### 2.2. Preparation of PP/GIC Composites

The PP/GIC composites were fabricated by melt mixing in an internal mixer (HTK-300, Hartek Tech Co. Ltd., Guangzhou, China). The PP pellets were dried at 80 °C for 4 h prior to melt mixing. The PP pellets were fed into the internal mixer and mixed with a rotor speed of 100 rpm for 10 min. After the PP pellets were fully molten, the GIC powder was added into the mixer. The PP/GIC composites were prepared at three different temperatures (180, 200, and 220 °C) with a weight ratio of 90:10. The as-prepared composites were denoted as PGt, where t represents the mixing temperature of the composites. The PP samples were also prepared for comparison at 180 °C with a rotor speed of 100 rpm. Then, the as-prepared samples were compression molded into sheets at a temperature of 180 °C for characterization.

### 2.3. Characterization

Scanning electron microscopy (SEM; EM-30N, Coxem, Daejeon, Korea) was used to characterize the microstructure of the PP/GIC composites. The molded sheets were cryofractured in liquid nitrogen to obtain specimens for characterization. All of the specimens were gold-sputtered and tested at an accelerating voltage of 15 kV.

Wide-angle X-ray diffraction (WAXD) measurements were carried out on an X-ray diffractometer (Mini Flex 600, Rigaku, Tokyo, Japan) at a scanning rate of 2°/min and a scanning step of 0.01°. The specimens with a size of 10 × 10 × 1 mm^3^ were tested at 2*θ* angle of 2–40°.

Dielectric property tests were performed on a dielectric analyzer (Agilent 4294A, Santa Clara, CA, USA). The specimens with a diameter of 25 mm and a thickness of 1 mm were cut from the molded sheets and tested in the frequency range 40–10^5^ Hz and at an operating AC voltage of 1 V.

A thermal constant analyzer (LW 9389, LonGwin, Hong Kong, China) was employed to measure the thermal conductivity of the prepared samples at 30 °C. Three specimens with a diameter of 25 mm and thickness of 1 mm were measured for each sample.

Crystallization and melting behaviors of the neat PP and PP/GIC composite samples were studied by using a differential scanning calorimeter (DSC; Netzsch 200F3, Selb, Germany). In order to erase any thermal history, the specimens were first heated to 190 °C at a rate of 10 °C/min and kept for 5 min. Then, the specimens were cooled to 30 °C and heated to 190 °C at a rate of 10 °C/min. Crystallinities (*X*_c_s) of the PP in the prepared samples were calculated by Equation (1) as below:

(1) $$X_{c}=\frac{∆H_{m}}{∆H_{m}^{0}\text{×}\omega }\text{×}100\%$$

where Δ*H*_m_, Δ$H_{m}^{0}$, and *ω* are melting enthalpy, melting enthalpy of 100% crystalline PP, and the weight fraction of the PP in the prepared samples, respectively. The value of Δ$H_{m}^{0}$ for the PP is 207 J/g [24].

A thermogravimetric analyzer (TGA; STA449C; Netzsch, Selb, Germany) was used to perform thermogravimetric analyses. Specimens were heated from room temperature to 600 °C at a heating rate of 10 °C/min under nitrogen atmosphere.

Tensile tests were performed by using a universal material testing machine (Instron 5566, Norwood, MA, USA) with a strain rate of 10 mm/min at room temperature. Five dumbbell-shaped specimens were measured for each sample.

## 3. Results and Discussion

### 3.1. Exfoliation and Dispersion of GICs

**Figure 1:**
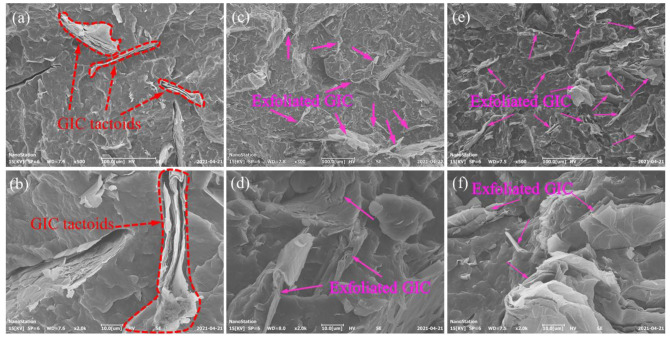
SEM micrographs of (**a**,**b**) PG180, (**c**,**d**) PG200, and (**e**,**f**) PG220 samples.

The SEM micrographs of the PP/GIC composite samples are illustrated in Figure 1. As can be seen in Figure 1a,b, some large GIC tactoids (marked by red dotted arrows) are presented in the PP matrix for the PG180 sample. With the increase in processing temperature, the size of the tactoids in the matrix becomes much smaller. Notably, for the PG220 sample (Figure 1e,f), the GICs are well exfoliated (as marked by the pink arrows) and dispersed in the PP matrix.

**Figure 2:**
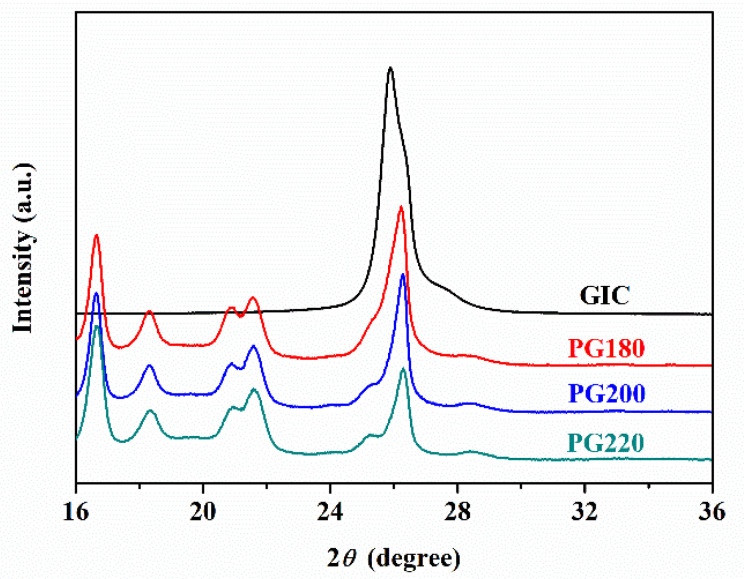
WAXD spectra of GIC, PG180, PG200, and PG220 samples.

Figure 2 presents WAXD patterns of the GICs and PP/GIC composite samples. For the PP/GIC composite samples, four characteristic peaks appear at 2*θ* = 13.9°, 16.7°, 18.3°, and 21.6°, which correspond to the (110), (040), (130), and (041) reflections of the α-phase, respectively. The characteristic peaks appearing at 2*θ*= 15.9° and 20.9° correspond to (300) and (301) reflections of the *β*-phase [25], respectively. Moreover, the GIC characteristic peak appears at about 2*θ* = 26.5° in all the prepared PP/GIC composites, and the intensity of the GIC characteristic peak in the composites decreases with the increase in processing temperature. Previous research [26,27] has shown that composites with well-exfoliated fillers present lower filler characteristic peak intensity in their spectrograms. The decrease in the GIC characteristic peak intensity in this work means that the GICs in the composites fabricated at a higher processing temperature possess a better exfoliation state.

### 3.2. Mechanism for GIC Intercalation and Exfoliation during Melt Mixing

**Figure 3:**
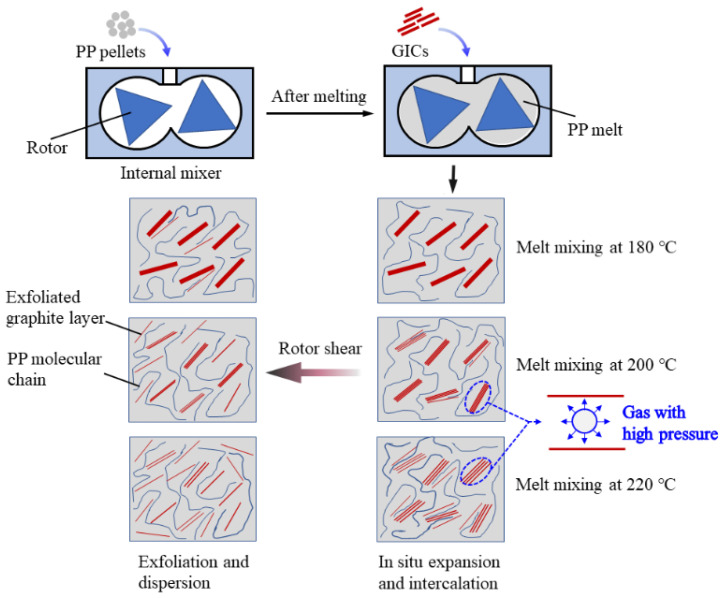
Schematics of underlying mechanism for in situ expansion, intercalation, exfoliation, and dispersion of GICs in PP matrix.

Figure 3 shows the mechanism of GIC exfoliation and dispersion in the PP matrix during the preparation of PP/GIC composites. The initial expansion temperature of the GICs used in this work is about 200 °C. For the PG180 composite, the GICs are not expandable at 180 °C. It is difficult for the PP molecular chain to intercalate into the GIC galleries. For the PG200 composite, the oxidized groups, such as acetic acid, nitric acid, and hydrogen peroxide, in the GICs decompose and release quantities of gases (mainly carbon dioxide and water vapor) with high pressure during melt mixing, which can expand GICs and increase their interlamellar spacing [28]. As a result, the GICs can be intercalated by the PP molecular chains easily and then exfoliated under the rotor shear. For the PG220 composite, the expansion ratio of the GICs further increases due to the higher processing temperature. Moreover, the mobility of the PP molecular chains can be enhanced due to the lower melt viscosity under the higher temperature, which may facilitate the intercalation of the PP molecular chains into the GIC galleries. Therefore, the PG220 sample exhibits better exfoliation and dispersion in the PP matrix.

### 3.3. Dielectric Properties

**Figure 4:**
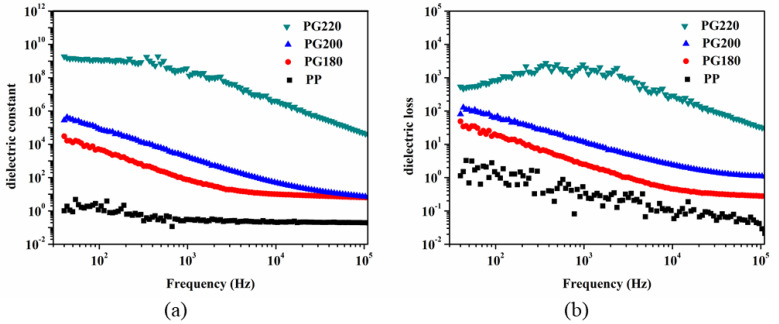
(**a**) Dielectric constant and (**b**) dielectric loss versus frequency for neat PP and PP/GIC composite samples.

The dielectric properties of the neat PP and PP/GIC samples were characterized at room temperature, and the results are illustrated in Figure 4. As displayed in Figure 4a, the neat PP sample shows a low dielectric constant over the entire test frequency range. For the PG180 sample, the dielectric constant significantly increases due to the addition of conductive GICs. With the increase in processing temperature, the dielectric constant of the PP/GIC composites further increases. Notably, the PG220 sample exhibits a dramatically improved dielectric constant over the entire test frequency range. Generally, the increase in the dielectric constant of polymer/conductive filler composites can be mainly ascribed to the interfacial polarization [29,30] and microcapacitor principle [31,32]. According to the Maxwell-Wagner-Sillars (MWS) mechanism [33,34], the GICs dispersed in the PP matrix can increase the interfacial polarization due to the large difference in dielectric constant between the GICs and PP matrix. With the increase in processing temperature, the dispersion of the GICs is clearly improved, which provides an extended surface that can be used to reinforce the MWS effect. Moreover, the spacing between the adjacent GICs in the PP matrix can be apparently reduced due to the improved exfoliation and dispersion of GICs, which can contribute significantly to the increase in the dielectric constant by forming a microcapacitor with GICs as electrodes and a very thin PP layer in between as dielectric. Notably, the dielectric constant of the PG220 sample at 10^3^ Hz is 1.3 × 10^8^, which is about six and five orders higher than that of the PG180 and PG200 samples, respectively. Simultaneously, the PP/GIC composites with a high dielectric constant are also accompanied by large dielectric loss. As can be seen in Figure 4b, the PG220 sample presents a dielectric loss of about 2000 at 10^3^ Hz, which is much higher than that of the other samples.

### 3.4. Thermal Properties

**Figure 5:**
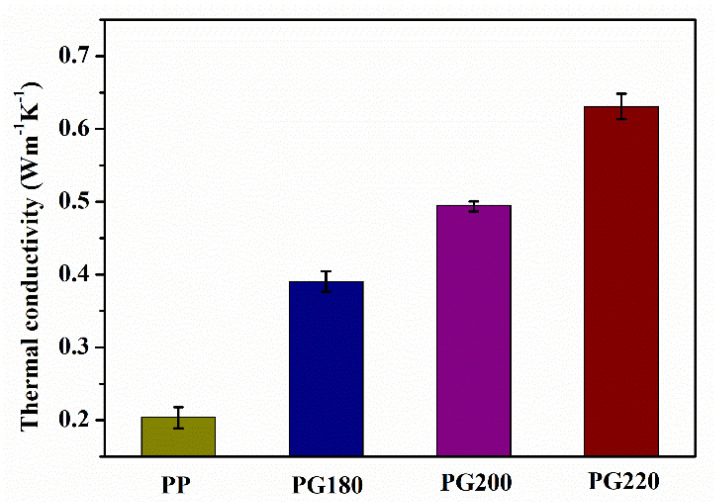
Thermal conductivities of neat PP and PP/GIC composite samples.

Figure 5 shows the thermal conductivity of the neat PP and PP/GIC composite samples. The neat PP sample exhibits a low thermal conductivity of about 0.20 Wm^−1^K^−1^. With the addition of 10 wt% GICs, the thermal conductivity increases to 0.39 Wm^−1^K^−1^ for the PP/GIC composite prepared at 180 °C. With the increase in processing temperature, the thermal conductivities of the PG200 and PG220 samples increase to 0.49 and 0.63 Wm^−1^K^−1^, respectively. Generally, the thermal conductivity of the polymeric composites is mainly dependent on the content of thermal conductive fillers. Composites with a higher filler volume fraction usually possess higher thermal conductivity. In this work, all the composites were prepared with a GIC content of 10 wt%. The PG220 sample exhibits a thermal conductivity that is 61.5% and 28.6% higher than that of the PG180 and PG200 samples, respectively. This is mainly ascribed to the fact that the well-dispersed GICs largely improve the phonon transport in the PG220 sample due to the reduced mean spacing between the adjacent GICs.

**Figure 6:**
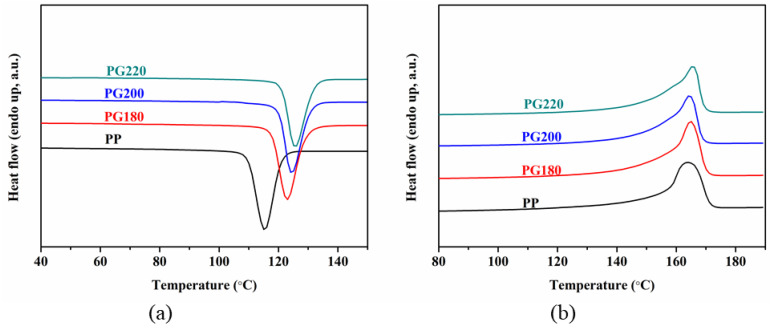
DSC (**a**) cooling and (**b**) heating curves for neat PP and PP/GIC composite samples.

DSC cooling and heating curves for the neat PP and PP/GIC composite samples are illustrated in Figure 6. As shown in Figure 6a, the neat PP sample exhibits a crystallization peak at 115.1 °C. With the addition of GICs, the crystallization peak temperature (*T*_c_) of the PP increases to 123.1 °C. This may be due to the fact that the GICs dispersed in the PP matrix can act as seeds for heterogeneous nucleation, which would accelerate the crystallization of PP. With the increase in processing temperature, the *T*_c_ of the composites further increases. Notably, PG220 presents the highest *T*_c_ of 126.1 °C. This implies that the GICs with better exfoliation and dispersion can further accelerate the crystallization of PP due to the increased nucleation point.

As illustrated in Figure 6b, the neat PP sample shows a melting peak at around 164.0 °C and exhibits a wide melting limit, which can be ascribed to the different integrity of the formed PP crystal. With the addition of GICs, especially with better exfoliation and dispersion state, the PP/GIC composite samples show narrowed melting limits and exhibit higher melting peak temperature (*T*_m_s) than that of the neat PP sample, which can be attributed to the formation of more uniform crystals in the composites induced by the GICs. The *X*_c_s of the neat PP and PP/GIC composite samples are calculated by Equation (1) and listed in Table 1. The neat PP sample shows an *X*_c_ of about 51.6%. For the PG180 sample, the addition of GICs clearly promotes the crystallization of the PP and increases the *X*_c_ to about 61.2%. With the increase in processing temperature, the *X*_c_ of the composites decreases gradually, which can be attributed to well-dispersed GICs with the high aspect ratio suppressing the growth of PP crystals in the composite samples by obstructing the mobility of the PP molecular chains.

**Figure 7:**
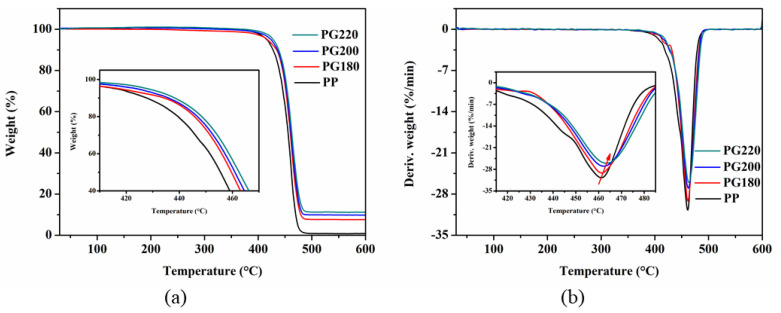
(**a**) TGA and (**b**) DTG thermograms for neat PP and PP/GIC composite samples.

The thermal stability of the neat PP and PP/GIC composite samples were evaluated using the TGA, and the results are illustrated in Figure 7. The thermal stability of the PP is effectively improved with the addition of GICs due to the “labyrinth effect” [35,36,37] caused by the GICs with a large specific surface area, which can absorb flammable organic volatiles or hinder their release and diffusion. With the increase in processing temperature, the thermal stability of the composites is continuously improved by the enhanced “labyrinth effect” due to the improved exfoliation and dispersion of GICs in the PP matrix, which leads to an increased, specific surface area. As can be seen in Figure 7b, the PG220 sample presents the highest temperature of about 364.5 °C for the maximum decomposition rate, which is 3.5 °C higher than that of the neat PP sample.

### 3.5. Tensile Properties

**Figure 8:**
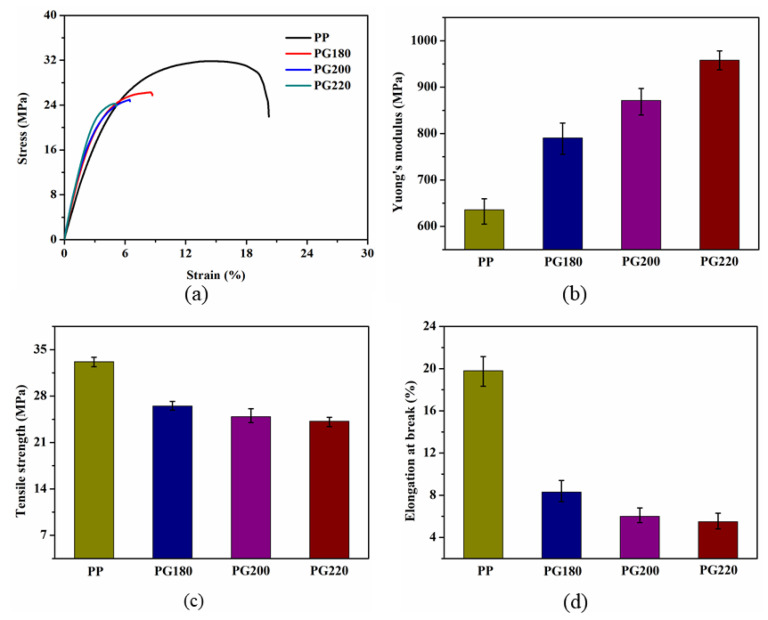
(**a**) Representative stress—strain curves, (**b**) Young’s modulus, (**c**) tensile strength, and (**d**) elongation at break of neat PP and PP/GIC composite samples.

The representative stress–strain curves of the PP and PP/GIC composites are shown in Figure 8a. It can be clearly observed that the PP/GIC composites show lower tensile strengths and elongations at break than those of the neat PP. The Young’s modulus, tensile strength, and elongation at break of all samples are obtained from the stress–strain curves, and the results are illustrated in Figure 8b-d along with their standard deviations. As can be seen in Figure 8b, the neat PP shows a Young’s modulus of about 635.7 MPa. With the addition of GICs, the Young’s modulus clearly increases due to the high intrinsic modulus of the stiff GICs [38]. The Young’s modulus of the composites is enhanced with the increase in processing temperature. The PG220 sample exhibits the highest Young’s modulus of about 958.2 MPa, which is 50.7%, 21.2%, and 10.0% higher than that of the neat PP, PG180, and PG200 samples, respectively. This can be attributed to the improved GIC dispersion in the PP matrix. Similarly, polymer composites with well-dispersed, stiff filler exhibit a higher Young’s modulus for PP reinforced with exfoliated graphite nanoplatelets [39].

As illustrated in Figure 8c, the tensile strength and elongation at the break of the neat PP are about 33.2 MPa and 19.8%, respectively. With the addition of GICs, the tensile strength of the composites decreases slightly with the increase in processing temperature. The tensile strength of the PG220 sample decreases to about 24.2 MPa. This can be ascribed to the poor interfacial adhesion between the PP and the GICs due to the poor wettability of GICs by the PP, which impedes the effective stress transfer across the PP matrix-GIC interface [25]. Then, the GICs dispersed in the PP matrix form stress concentration points leading to stress or catastrophic failure. Moreover, the elongation at break notably decreases, especially for the composites prepared at a higher temperature. The elongation at break of the PG220 sample decreases substantially to about 5.5%.

## 4. Conclusions

The dispersion of GICs is notably improved by the increase in processing temperature. The GICs can be intercalated by the PP molecular chains easily due to the increased interlamellar spacing due to the in situ expansion at above 200 °C and then exfoliated under the rotor shear. As a result, the dielectric constant and thermal conductivity of the PP/GIC composite samples (with a GIC content of 10 wt%) are significantly improved. The PG220 sample exhibits a dielectric constant of about 1.3 × 10^8^ at 10^3^ Hz, which is about six and five orders higher than that of the PG180 and PG200 samples, respectively. Simultaneously, the PG220 sample exhibits a thermal conductivity of 0.63 Wm^−1^K^−1^, which is 61.5% and 28.6% higher than that of the PG180 and PG200 samples, respectively. The well-dispersed GICs in the PP matrix would accelerate the crystallization of PP due to the increased nucleation point. However, its high aspect ratio would suppress the growth of the PP crystal by hampering the mobility of the PP chains, leading to a decreased *X*_c_. The thermal stability of the composites is enhanced by the improved dispersion of GICs in the PP matrix because of the “labyrinth effect” caused by the well-dispersed GICs. The Young’s modulus of the PP/GIC composites is notably enhanced with the increase in processing temperature. The PG220 sample exhibits a Young’s modulus of about 958.2 MPa, which is 50.7%, 21.2%, and 10.0% higher than that of the neat PP, PG180, and PG200 samples, respectively. This can be attributed to the high intrinsic modulus and improved dispersion of the stiff GICs. However, the elongation at the break of the PG220 sample decreases substantially to about 5.5%.

## Figures and Tables

**Table 1:** DSC thermal performance parameters for neat PP and PP/GIC composite samples.

Sample	*T*_c_ (°C)	Δ*H*_m_ (J/g)	*T*_m_ (°C)	*X*_c_ (%)
PP	115.4	106.8	164.0	51.6
PG180	123.0	114.1	164.5	61.2
PG200	124.5	101.2	164.8	54.3
PG220	125.6	96.7	165.6	51.9

## Data Availability

The data presented in this study are available on request from the corresponding author.
